# Exploring shared neural substrates underlying cognition and gait variability in adults without dementia

**DOI:** 10.1186/s13195-023-01354-y

**Published:** 2023-11-27

**Authors:** Seonjeong Byun, Hyang Jun Lee, Jun Sung Kim, Euna Choi, Subin Lee, Tae Hui Kim, Jae Hyoung Kim, Ji Won Han, Ki Woong Kim

**Affiliations:** 1 Department of Neuropsychiatry, College of Medicine, Uijeongbu St Mary’s Hospital, The Catholic University of Korea, Seoul, Republic of Korea; 2 Department of Neuropsychiatry, Seoul National University Bundang Hospital, 82 Gumiro 173 Beongil, Bundanggu, Seongnamsi, Gyeonggido 463-707 Republic of Korea; 3 Institute of Human Behavioral Medicine, Seoul National University Medical Research Center, Seoul, Republic of Korea; 4 Department of Brain and Cognitive Sciences, Seoul National University College of Natural Sciences, Seoul, Republic of Korea; 5 Laboratory for Imaging Science and Technology, Department of Electrical and Computer Engineering, Seoul National University, Seoul, Republic of Korea; 6 Department of Psychiatry, Yonsei University Wonju Severance Christian Hospital, Wonju, Republic of Korea; 7 Department of Radiology, Seoul National University Bundang Hospital, Seongnam, Republic of Korea; 8 Department of Radiology, Seoul National University College of Medicine, Seoul, Republic of Korea; 9 Department of Psychiatry, Seoul National University College of Medicine, Seoul, Republic of Korea

**Keywords:** Gait, Tri-axial accelerometer, Cognitive impairment, Digital biomarker, Shared neural network

## Abstract

**Background:**

High gait variability is associated with neurodegeneration and cognitive impairments and is predictive of cognitive impairment and dementia. The objective of this study was to identify cortical or subcortical structures of the brain shared by gait variability measured using a body-worn tri-axial accelerometer (TAA) and cognitive function.

**Methods:**

This study is a part of a larger population-based cohort study on cognitive aging and dementia. The study included 207 participants without dementia, with a mean age of 72.6, and 45.4% of them are females. We conducted standardized diagnostic interview including a detailed medical history, physical and neurological examinations, and laboratory tests for cognitive impairment. We obtained gait variability during walking using a body-worn TAA along and measured cortical thickness and subcortical volume from brain magnetic resonance (MR) images. We cross-sectionally investigated the cortical and subcortical neural structures associated with gait variability and the shared neural substrates of gait variability and cognitive function.

**Results:**

Higher gait variability was associated with the lower cognitive function and thinner cortical gray matter but not smaller subcortical structures. Among the clusters exhibiting correlations with gait variability, one that included the inferior temporal, entorhinal, parahippocampal, fusiform, and lingual regions in the left hemisphere was also associated with global cognitive and verbal memory function. Mediation analysis results revealed that the cluster’s cortical thickness played a mediating role in the association between gait variability and cognitive function.

**Conclusion:**

Gait variability and cognitive function may share neural substrates, specifically in regions related to memory and visuospatial navigation.

**Supplementary Information:**

The online version contains supplementary material available at 10.1186/s13195-023-01354-y.

## Background

The large public health burden of dementia and the absence of promising disease-modifying therapy highlight the need for early identification of those at risk for cognitive decline or dementia to prevent and/or delay its onset [1]. Emerging evidence indicates that changes in gait can be a promising biomarker for the early identification of individuals at high risk of dementia. Several previous studies demonstrate older adults with slower gait speed were at higher risk of accelerated cognitive decline and incident mild cognitive impairment (MCI) or dementia [2, 3]. A multi-national epidemiological study revealed that motoric cognitive risk syndrome accompanied by cognitive complaints and slower gait speed (MCR-S) increased the risk of dementia, which affects about 10% in older adults [4]. Gait variability, the fluctuation of a gait measure from one step to the next, was also strongly associated with the risk of cognitive decline, MCI, and dementia [5–7]. Motoric cognitive risk syndrome accompanied by cognitive complaints and higher gait swing time variability (MCR-SWV) was associated with a risk of cognitive decline [8].

However, the types of cognitive disorders and cognitive impairments predicted based on slower gait speed differed from those predicted based on higher gait variability. MCR-S predicted the risk of vascular dementia, but not AD [9]. In contrast, higher gait variability accurately distinguished Alzheimer’s disease (AD) from other neurodegenerative and cognitive conditions [10]. MCR-S predicted incident impairments in language, whereas MCR-V predicted those in memory and visuospatial function [8]. These results indicate that increased gait variability may be attributable to different neural substrates from decreased gait speed.

Higher gait variability is associated with lower gray matter integrity and neuronal metabolism of the hippocampus, lower gray matter integrity of the anterior cingulate gyrus, and decreased parietal gray matter volume [11–13]. Since patients with AD exhibited neurodegenerative changes in some of these brain regions [14, 15], increased gait variability and cognitive decline may have common neural substrates. However, the neural substrates shared by high gait variability and cognitive decline have not been directly investigated yet. While previous studies have explored the neural substrates associated with gait variability, many have focused on pre-selected regions of interest (ROIs) [11, 16, 17]. This approach may be limiting, as the neural networks involved in gait are scattered throughout various regions of brain. Additionally, earlier researches have primarily concentrated on volume measurements, whereas cortical thickness might provide a more sensitive indicator for detecting cortical degeneration [18]. Furthermore, However, some previous studies have used force plates that were limited to lengths of 12–14 ft, which may not provide the minimum number of consecutive steps needed for more reliable gait variability measurements [19]. Therefore, there is a need to utilize tools that are capable of measuring a sufficiently large number of consecutive steps and come with relatively low spatial constraint and cost.

The aim of the present cross-sectional study was to identify cortical or subcortical structures of the brain shared by gait variability measured using a body-worn tri-axial accelerometer (TAA) and cognitive function.

## Methods

### Participants

This study is embedded in the Korean Longitudinal Study on Cognitive Aging and Dementia (KLOSCAD), a population-based prospective multicenter cohort study of Koreans aged 60 years and older. The KLOSCAD was launched in 2009 and was followed up biennially until 2020 [20]. We included 207 of the 232 individuals who simultaneously completed gait evaluation and brain MRI in the KLOSCAD cohort in the final analysis after excluding the following conditions: (1) dementia or major psychiatric disorders according to the Diagnostic and Statistical Manual of Mental Disorders (4th ed., text revision) criteria; (2) major neurologic disorders including Parkinson’s disease, brain tumor, or stroke; (3) history of traumatic brain injury; (4) Tinetti Performance Oriented Mobility Assessment—Gait subscale (POMA-G) score of ≤ 10; (5) one or more cardinal signs (bradykinesia, tremor, rigidity) or two or more non-cardinal signs of parkinsonism according to the Unified Parkinson’s Disease Rating Scale Part III (UPDRS).

All participants provided written informed consent themselves or via their legal guardians. The present study was approved by the Institutional Review Board of the Seoul National University Bundang Hospital.

### Assessment of cognition and medical conditions

Geriatric psychiatrists performed a standardized diagnostic interview that included a detailed medical history, physical and neurological examinations, and laboratory tests on each subject using the Korean version of the Consortium to Establish a Registry for Alzheimer’s Disease Assessment Packet Clinical Assessment Battery (CERAD-K-C) and Mini International Neuropsychiatric Interview [21, 22]. They evaluated the comorbidity and vascular burdens using the Cumulative Illness Rating Scale (CIRS) and Modified Hachinski Ischemic Score, respectively, and determined whether degenerative arthritis of the spine and/or lower extremities was present using the musculoskeletal category of the CIRS. They evaluated Parkinsonian symptoms and gait disturbances using the UPDRS and POMA-G. The maximum UPDRS score is 108, and higher scores indicate more severe Parkinsonian motor symptoms. The maximum POMA-G score is 12 and higher scores indicate improved gait performance.

Trained neuropsychologists or research nurses performed neuropsychological assessments, including the Korean version of the Consortium to Establish a Registry for Alzheimer’s Disease Neuropsychological Assessment Battery (CERAD-K-N) [21], Korean version of the Frontal Assessment Battery [23], and Digit Span Test. The CERAD-K-N consists of nine neuropsychological tests, including the Categorical Fluency Test (CFT), Modified Boston Naming Test (mBNT), Mini Mental Status Examination (MMSE), Word List Memory Test (WLMT), Constructional Praxis Test (CPT), Word List Recall Test (WLRT), Word List Recognition Test (WLRcT), Constructional Recall Test (CRT), and Trail Making Test A. We calculated the CERAD-K total scores (CERAD-TS) by summing the CFT, mBNT, WLMT, WLRT, WLRcT, and CRT scores. We defined the Verbal Memory Score (VMS) as the weighted average of the WLMT, WLRT, and WLRcT scores. The CERAD-TS and VMS range from 0 to 100 and 0 to 30, respectively, and higher scores indicate better cognitive function.

Research nurses asked the participants to self-perform the Korean version of Geriatric Depression Scale (GDS) to evaluate the severity of depressive symptoms [24].

### Gait assessment

We measured the temporal gait variability because temporal parameters were more affected by dementia-related gait parameters than spatial parameters. Additionally, temporal parameters were associated with AD pathology; however, spatial parameters were not [25]. We used steps instead of strides to measure temporal gait variability because the gait variability from the left and right steps combined was more reliable than using strides [26].

We previously measured variations in the step time of each participant using a TAA (FITMETER® [FitLife Inc., Suwon, Korea] or ActiGraph® [SMD solution, Seoul, Korea]) placed over the center of body mass (CoM) [19]. The inertial measurement units (IMU) were hexahedrons (35 × 35 × 13 mm [14 g]/30 × 40 × 10 mm [17 g]) with smooth edges and a digital TAA (BMA255, BOSCH, Germany) and gyroscope (BMX055, BOSCH, Germany). They could measure tri-axial acceleration and velocity up to ± 8 g (with a resolution of 0.004 g/0.00024 g) and ± 1000°/s (with a resolution of 0.03°/s) at 250 Hz, respectively. We attached an IMU to each participant at the 3rd–4th lumbar vertebrae using Hypafix. We asked each participant to walk back and forth three times on a 14-m flat straight walkway at a comfortable self-selected pace and start turning after passing the 14 m line. To measure steady-state walking, we analyzed the data of the central 10 m of the 14 m-walk after the 2 m-walks prior to the start and each turn were eliminated. We calculated step time variability from the vertical acceleration data using the method described by Zijlstra and Hof [i.e., % coefficient of variation (% CV) of step time = (standard deviation of step time/mean step time) × 100] [27]. In the present study, we used the natural log transformation of %CV of step time as the gait variability as the %CV of step time was not normally distributed. The detailed methods of signal processing and gait variability calculation are described elsewhere [19].

We also measured the leg length, i.e., the distance between the anterior superior iliac spine and lateral malleolus, as a covariate because leg length is associated with spatiotemporal gait parameters.

### Acquisition and preprocessing of MRI

We obtained three-dimensional structural T1-weighted spoiled gradient echo magnetic resonance (MR) images of the participants within a year after their clinical and neuropsychological assessments using a 3.0 Tesla GE SIGNA Scanner (GE Healthcare; Milwaukee, WI) in Digital Imaging and Communications in Medicine format with the following parameters: acquired voxel size, 1.0 × 0.5 × 0.5 mm^3^; 1.0 mm sagittal slices with no inter-slice gap; echo time, 3.68 ms; repetition time, 25.0 ms; number of excitations, 1; flip angle, 90°; field of view, 240 × 240 mm; 175 × 240 × 240 matrix in the x-, y-, and z- dimensions. We bias-corrected the T1 images to remove intensity inhomogeneity artifacts using Statistical Parametric Mapping software (version 8, SPM8; Wellcome Trust Centre for Neuroimaging, London; http://www.fil.ion.ucl. ac.uk/spm). We then resliced the bias-corrected T1 images into isotropic voxels (1.0 × 1.0 × 1.0 mm^3^).

We performed cortical reconstruction and volumetric segmentation using FreeSurfer v6.0 (http://surfer.nmr.mgh.harvard.edu/). We smoothed thickness maps with a 10 mm full-width half-maximum (FWHM) Gaussian kernel before performing statistical analysis. Based on gyral and sulcal anatomy, we segmented the cortex into 34 gyral regions per hemisphere (13 frontal, 9 temporal, 4 occipital, 7 parietal, and the insula), using the Desikan–Killiany Atlas [28].

### Statistical analyses

To examine the association of gait variability with cognitive function measures (CERAD-TS and VMS), we created a multivariate general linear model (GLM) adjusted for age, sex, education, GDS, CIRS, leg length, and presence of arthritis using the linear model function of the Stats package in R version 3.3.2 software (R Foundation for Statistical Computing).

To determine the association between gait variability and cortical thickness, we performed vertex-wise analyses using the FreeSurfer QDEC module (Query, Design, Estimate, Contrast (http://surfer.nmr.mgh.harvard.edu)), which allows users to perform inter-subject/group averaging and inference using the general linear model on morphometric data. We applied corrections for multiple comparisons using the built-in Monte Carlo simulation at a threshold of *p* = 0.05, a cluster-wise correction that controls for the rate of false positive clusters. In QDEC, we used a GLM with each gait parameter as the continuous predictor. Age and estimated total intracranial volume (eTIV) were set as nuisance variables within the different offset and slope design matrix. As the number of covariates in QDEC is limited, we exported each participant’s cortical thickness in the identified clusters to R to assess whether the associations withstood correction for confounding factors. To do so, we created a ROI for each cluster that was significantly associated with gait variability. We mapped this normalized ROI to each participant to generate a mean thickness value for that ROI for each participant. We performed additional linear model analyses using the mean cortical thickness of the ROIs as dependent variables and gait variability as an independent variable. We corrected for age, sex, education level, GDS, CIRS, leg length, presence of arthritis, and eTIV.

To determine the association between gait variability and the volumes of the subcortical grey matter structures (caudate, putamen, globus pallidus, thalamus, and nucleus accumbens), amygdala, hippocampus, and cerebellum, we created a multivariate GLM adjusted for age, sex, education, GDS, CIRS, leg length, presence of arthritis, and eTIV. False discovery rate correction was applied to correct for multiple comparisons. Eight ROIs were selected a priori from each hemisphere based on their known associations with gait control.

To determine the association between cognitive function measures and the cortical thickness and subcortical volume of the structures associated with the gait variability, we created a multivariate GLM that adjusted for age, sex, education, GDS and CIRS scores, and eTIV.

Lastly, we analyzed the mediation effect of the cortical thickness and subcortical volume of clusters that were significantly associated with both gait variability and cognitive function on the association between these factors (VMS, CERAD-TS) using the PROCESS macro developed for SPSS [29]. We performed parallel mediation analyses separately for each cognitive assessment using 5000 bootstrapped samples. In these analyses, we adjusted for sex, age, education, GDS, CIRS, and eTIV. Path a represents the effect of gait variability on the neuroimaging measures, whereas path b represents the effect of neuroimaging measures on cognition. Paths c and c’ indicate the total and direct effects of gait variability on cognition, respectively. The indirect effect (path a × b) measures the effect of gait variability on cognition via the cluster cortical thickness or subcortical volume. A significant indirect effect is indicated by 95% confidence intervals that do not include the value of 0.

## Results

In the present study, both men and women were included, with men comprising 54.6% of the 207 participants. As summarized in Table 1, men were more educated (mean difference, 3.58 years; *t*, 6.82; *p* < 0.001), had longer legs (mean difference, 5.36 cm; *t*, 6.43; *p* < 0.001), and exhibited higher CERAD-TS (mean difference, 3.11 scores; *t*, 2.00; *p* = 0.047) than women. Although neither men nor women exhibited signs of a depressive disorder, women had more depressive symptoms than men, as evidenced by the mean difference in GDS scores (mean difference, 3.16; *t*, 4.09; *p* < 0.001). Degenerative arthritis of the spine or lower limbs was less prevalent in men than in women (*χ*^2^, 23.50; *p* < 0.001).

**Table 1:** Characteristics of participants (*N* = 207)

	All (*N* = 207)	Male (*N* = 113)	Female (*N* = 94)	p*
Age at *MRI (years)	72.7 ± 6.7	73.0 ± 6.9	72.2 ± 6.5	0.375
Education (years)	13.0 ± 4.1	14.6 ± 3.7	11.0 ± 3.8	< 0.001
Leg length (cm)	84.2 ± 6.7	86.7 ± 6.8	81.3 ± 5.2	< 0.001
Gait variability (ln % CV^†^)	0.9 ± 0.3	0.9 ± 0.3	0.9 ± 0.2	0.313
^‡^GDS (points)	7.9 ± 5.7	6.5 ± 5.3	9.6 ± 5.7	< 0.001
^§^CIRS (points)	7.1 ± 3.3	7.2 ± 3.6	6.9 ± 2.8	0.575
^¶^MHIS (points)	0.8 ± 1.2	0.9 ± 1.4	0.7 ± 0.8	0.283
Existence of arthritis (%)	29.0	15.0	45.7	< 0.001
^#^CERAD-TS (points)	76.8 ± 10.9	78.2 ± 9.2	75.1 ± 12.5	0.047
**VMS (points)	21.7 ± 4.1	21.8 ± 4	21.7 ± 4.3	0.859

*MRI Magnetic resonance imaging, ^†^*CV* Coefficient of variance, ^‡^*GDS* Geriatric Depression Scale, ^§^*CIRS* Cumulative Illness Rating Scale, ^¶^*MHIS* Modified Hachinski Ischemic Score, ^#^*CERAD-TS* Consortium to Establish a Registry for Alzheimer’s Disease Assessment Packet Neuropsychological Assessment Battery total score, **VMS Verbal Memory Score

*Student’s *t*-test was performed for continuous variables (presented as mean ± standard deviation) and the chi-square test for categorical variables (presented as %)

^†^Natural log transformation of %CV of step time was used as gait variability since %CV of step time was not normally distributed

Higher gait variability was associated with lower CERAD-TS (*t*_,_ − 3.56; *p* < 0.001) and VMS (*t*, − 3.44; *p* < 0.001) in the multivariate GLM adjusted for age, sex, education, GDS, CIRS, leg length, and presence of arthritis (*R*^2^, 0.260; *F*_6,200_, 11.73; *p* < 0.001 for CERAD-TS and *R*^2^, 0.022; *F*_6,200_, 9.16; *p* < 0.001 for VMS).

**Fig. 1:**
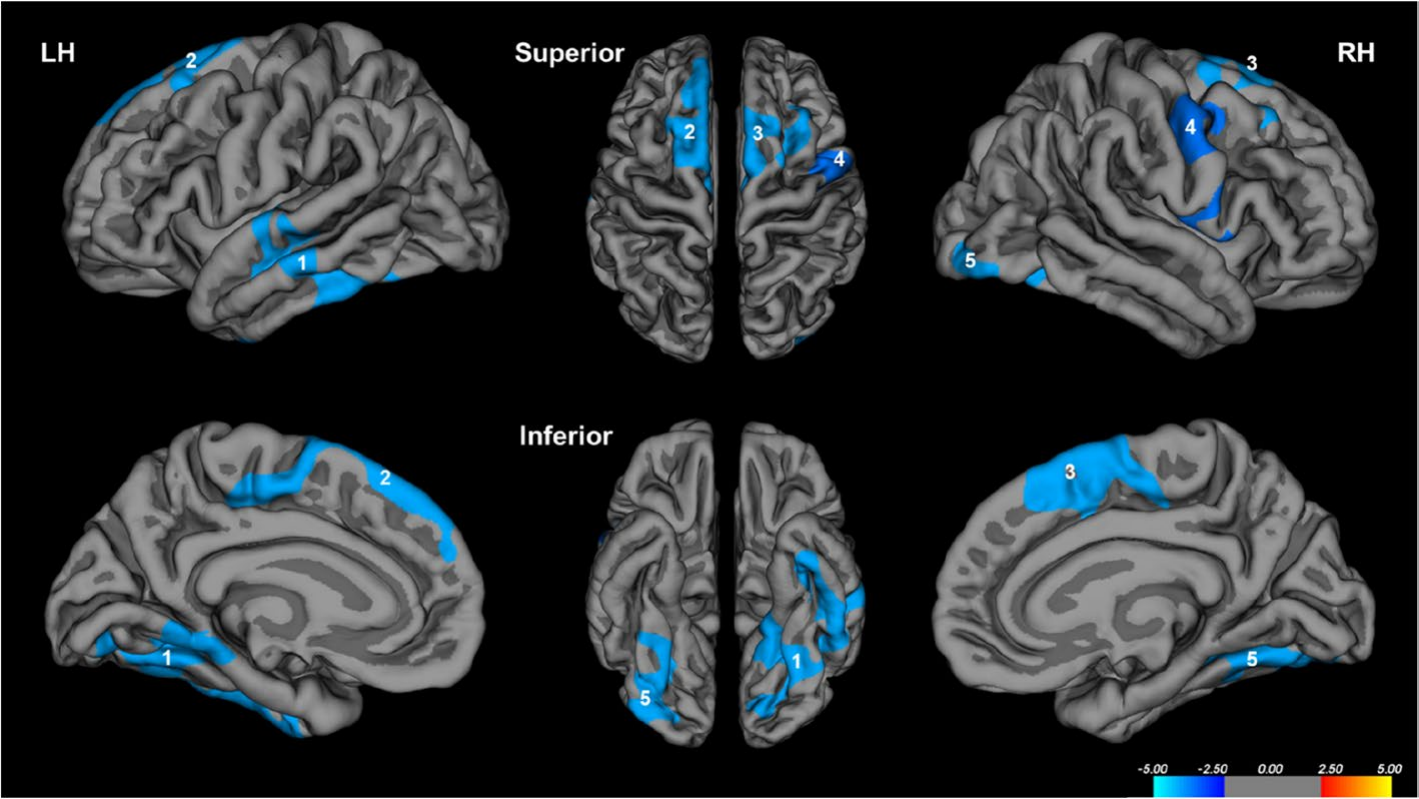
Cortical thickness and gait variability in non-demented older adults. Analyses were adjusted for age and eTIV. Colors represent –log10(*p* value). Blue clusters represent a negative correlation with gait variability. Numbers refer to the entire cluster. eTIV, estimated total intracranial volume; LH, left hemisphere; RH, right hemisphere

As summarized in Table 2 and Fig. 1, higher gait variability was associated with reduced cortical thickness in five regions (2 and 3 clusters in the left and right hemispheres, respectively) in the vertex-wise analysis. However, the volume of these specific clusters was not associated with gait variability in any of the clusters. In the left hemisphere, one cluster (LH1) included the inferior temporal cortex, covering portions of the middle, and superior temporal cortices. This cluster extended medially to include the entorhinal, and para-hippocampal cortices, as well as posteriorly to include the fusiform gyrus and lingual cortex (*p* = 0.0001). The other cluster (LH2) included the superior frontal gyrus, which contains the supplementary motor area and medial frontal gyrus. It also covered a part of the paracentral lobule (*p* = 0.0001). In the right hemisphere, one cluster (RH1) included the superior frontal gyrus, which primarily includes the supplementary motor area, medial frontal gyrus, and paracentral lobule. This cluster extended laterally to part of the caudal and rostral middle frontal gyri (*p* = 0.0001). Another cluster (RH2) included the precentral gyrus and extended anteriorly to include part of the caudal middle frontal cortex and inferiorly to the pars opercularis (*p* = 0.0004). The other cluster (RH3) included the fusiform gyrus and lateral occipital cortex (*p* = 0.0001). As shown in Table 3, these associations remained significant when sex, education level, GDS, CIRS, leg length, and presence of arthritis were adjusted.

**Table 2:** Vertex-wise analyses of gait variability and cortical thickness (*N* = 207)

Clusters	Cluster size (mm^2^)	Talairach coordinates (*x*, *y*, *z*)	Number of vertices within cluster	Cluster-wise p*
Left hemisphere				
Temporal/fusiform (LH1)	4460.81	− 53.1, − 24.0, − 4.0	7678	0.0001
Superior frontal/paracentral (LH2)	1766.48	− 6.6, 33.8, 49.8	3417	0.0001
Right hemisphere				
Superior frontal/paracentral (RH1)	2289.14	11.0, 14.6, 62.2	4455	0.0001
Fusiform/lingual (RH2)	1792.45	34.3, − 73.5, − 12.0	2823	0.0001
Precentral (RH3)	1723.35	40.2, − 10.9, 42.6	3519	0.0004

*Analyses were corrected for multiple comparisons using the built-in Monte Carlo simulation at a threshold set at *p* < 0.05, a cluster-wise correction that controls for the rate of false positive clusters

**Table 3:** Regression analyses of cortical thickness and gait variability

Clusters	B	SE	*t*	*p*	β
Left hemisphere					
Temporal/fusiform (LH1)					
Unadjusted	− 0.196	0.038	− 5.168	< 0.001	− 0.329
Adjusted^a^	− 0.177	0.038	− 4.685	< 0.001	− 0.297
Superior frontal/paracentral (LH2)					
Unadjusted	− 0.219	0.048	− 4.543	< 0.001	− 0.302
Adjusted^a^	− 0.209	0.049	− 4.264	< 0.001	− 0.289
Right hemisphere					
Superior frontal/paracentral (RH1)					
Unadjusted	− 0.225	0.045	− 5.010	< 0.001	− 0.330
Adjusted^a^	− 0.211	0.045	− 4.668	< 0.001	− 0.309
Fusiform/lingual (RH2)					
Unadjusted	− 0.222	0.046	− 4.801	< 0.001	− 0.309
Adjusted^a^	− 0.213	0.046	− 4.578	< 0.001	− 0.296
Precentral (RH3)					
Unadjusted	− 0.225	0.044	− 5.065	< 0.001	− 0.333
Adjusted^a^	− 0.214	0.044	− 4.894	< 0.001	− 0.316

^a^ Adjusted for age and total intracranial volume. Adjusted model additionally adjusted for sex, education level, geriatric depression scale score, cumulative illness rating scale score, leg length, and presence of arthritis

**Fig. 2:**
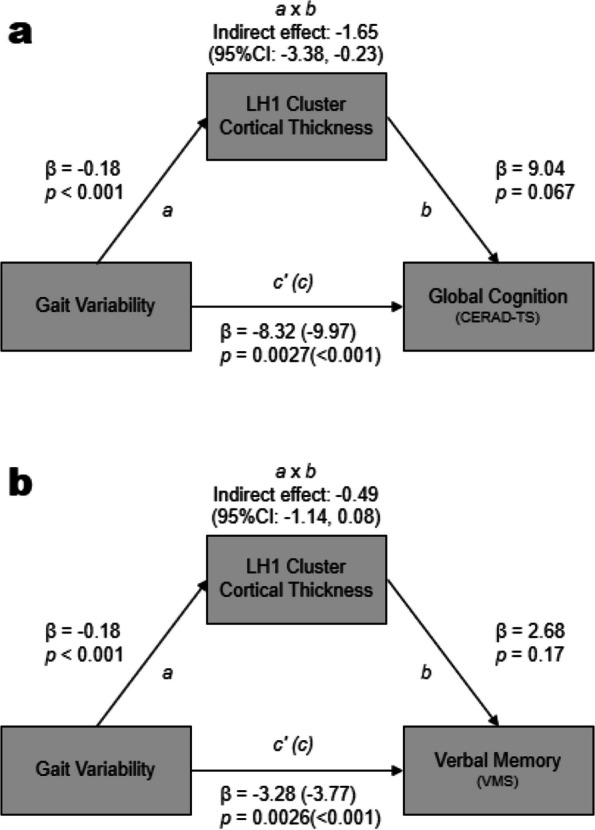
Cortical thickness of LH1 cluster mediates the effect of gait variability on **a** CERAD-TS and **b** VMS. LH1 cluster: a cluster that includes part of the temporal, fusiform, and lingual gyrus; VMS, Verbal Memory Score; CERAD-TS, Consortium to Establish a Registry for Alzheimer’s Disease Assessment Packet Neuropsychological Assessment Battery total score; GDS, Geriatric Depression Scale; CIRS, Cumulative Illness Rating Scale; eTIV, estimated total intracranial volume. Parallel mediation analyses adjusted for sex, age, education, GDS, CIRS, and eTIV

Cortical thinning of the LH1 was associated with lower CERAD-TS and VMS. This remained the case when age, sex, education level, GDS, CIRS, leg length, presence of arthritis, and eTIV were adjusted. However, the cortical thickness of other clusters was not associated with CERAD-TS and VMS (Table 4). In the mediation analyses, the cortical thickness of LH1 mediated the association between gait variability and CERAD-TS (indirect effect, − 1.65; SE, 0.79, bias-corrected 95% confidence interval [− 3.38, − 0.23] (Fig. 2A)) and explained 17% of the total effect of gait variability on CERAD-TS. However, the mediating role of the cortical thickness of LH1 in the association between gait variability and VMS was not statistically significant (indirect effect, − 0.49; SE, 0.31; bias-corrected 95% confidence interval [− 1.14, 0.08] (Fig. 2B)).

**Table 4:** Associations between cortical regions associated with gait variability and cognitive function (*N* = 207)*

	*CERAD-TS				^†^VMS			
	*B*	SE	*t*	*p*	*B*	^‡^SE	*t*	*p*
Left hemisphere								
Temporal/fusiform (LH1)	14.09	4.74	2.97	0.003	4.71	1.86	2.53	0.01
Superior frontal/paracentral (LH2)	3.62	3.80	0.95	0.34	1.62	1.48	1.09	0.28
Right hemisphere								
Superior frontal/paracentral (RH1)	3.35	4.10	0.82	0.42	1.60	1.60	1.00	0.31
Fusiform/lingual (RH2)	6.80	3.97	1.72	0.09	1.58	1.55	1.02	0.31
Precentral (RH3)	8.00	4.22	1.90	0.06	2.92	1.65	1.77	0.08

*Adjusted for sex, age, education, geriatric depression scale score, cumulative illness rating scale score, leg length, presence of arthritis, and estimated total intracranial volume

*CERAD-TS Consortium to Establish a Registry for Alzheimer’s Disease Assessment Packet Neuropsychological Assessment Battery total score, ^†^*VMS* Verbal Memory Score, ^‡^*SE* Standard error

## Discussion

This study found that higher gait variability was associated with poorer global cognition and verbal memory in non-demented older adults, which aligns with our previous work on cognitively normal older adults [30]. One of the standout findings of this study is that cortical thinning in a cluster involving the inferior temporal, entorhinal, parahippocampal, fusiform, and lingual regions in the left hemisphere is closely linked to higher gait variability and correlates with lower verbal memory and global cognitive function.

The current study found that higher step time variability was associated with thinner GM in the prefrontal, supplementary motor, and paracentral lobules in both hemispheres and the superior temporal, middle temporal, and inferior temporal areas in the left hemisphere. The motor cortex exhibits significantly thinner cortical thickness with higher temporal gait variability in the present study, which is consistent with the results of previous studies [31, 32]. More specifically, we identified cortical thinning of the paracentral lobule, i.e., the medial continuation of primary motor and sensory gyri, which controls lower limb movement. We also found that the thinning of the medial frontal gyri, including the supplementary and pre-supplementary motor areas, was associated with higher temporal gait variability. These findings suggest that the primary motor cortex is involved in the execution phase (i.e., converting motor programs into movements), but other frontal areas are also involved in planning and programming and may influence temporal gait variability. Additionally, other non-frontal regions that play important roles in the visual network such as the fusiform gyrus, left parahippocampal and inferior temporal regions, lingual gyri, and right lateral occipital cortex also influenced temporal gait variability. These regions are involved in visual processing, visual perception, and spatial orientation and navigation [33–35]. Dynamic instability may be better explained by cerebral cortical misprocessing than abnormal subcortical gait control.

To the best of our knowledge, this is the first study to directly demonstrate the gait-cognition relationship through a shared neural network in an older, non-demented population. We combined exploratory mapping and a priori ROI-based measurement techniques by performing an exploratory analysis of cortical thickness throughout the cortical mantle to map the “cortical signature” of regional thinning correlated with gait variability. We then used this map to generate ROIs to simultaneously determine a priori whether regional cortical thinning was correlated with poorer cognitive functions. We discovered that cortical thinning of the cluster including the entorhinal, parahippocampal, fusiform, lingual, and inferior temporal regions in left hemisphere that is linked to gait variability was also correlated with lower VMS and CERAD-TS. The medial temporal cortex, including the entorhinal and parahippocampal cortices, is related episodic memory and is one of the first regions to exhibit neurodegeneration in patients with AD [36]. Also, the network coves and entorhinal, parahippocampal, and fusiform areas is involved in visuospatial navigation and imagination of the visual environment, which is necessary for locomotion [37]. Recent studies have also accentuated the specialized role of the medial temporal cortex in encoding locomotion speed through “speed cells” [38, 39]. Using mediation analysis, we confirmed that the cortical thickness of the cluster, including the entorhinal, parahippocampal, fusiform, lingual, and inferior temporal areas in left hemisphere mediates the association between CERAD-TS and gait variability. This accounted for 17% of the total effect of gait variability on CERAD-TS. Our findings suggest that gait variability and cognitive function rely on shared neural systems that are the first to be affected by pathological aging, such as AD. In a subgroup of individuals with mild cognitive impairment, regions in the left hemisphere including the inferior temporal, fusiform, and lingual areas consistently showed an association with gait variability (Additional file 1: Figure S1). A recent multisite cross-sectional study on older adults with varying neurodegenerative conditions revealed that high gait variability distinguished AD from other neurodegenerative and cognitive conditions. Taking a step further from the results, the present study showed that neurodegenerative changes in widespread cerebral regions, measured by cortical thinning, may manifest as increased gait variability at an earlier stage than can indicate a clinical diagnosis of dementia. Our results also suggest that gait variability obtained using a body-worn TAA may be a digital biomarker of neurodegenerative diseases such as AD. TAA sensors are commonly integrated into widely available smartphones and smartwatches, and if needed, standalone TAA hardware is also relatively low cost. This cost-effectiveness, along with the absence of the need for specialized personnel for administration, makes it particularly suited for large-scale screenings, including in low- and middle-income countries. Its properties of being free from time and space constraints and low cost makes it potentially usable in a clinical setting or clinical trials, especially in non-face-to-face environments. By utilizing this scalable screening technology for early identification of at-risk populations, targeted interventions—ranging from physical exercise regimens to nutritional modifications, cognitive and brain reserve augmentation, and cardiovascular risk management—can be promptly initiated. Our results also highlight the importance of examining comprehensive metrics of gait beyond simple gait speed measurement.

The current study also revealed that the cortical thickness of the entorhinal and parahippocampal cortices was associated with gait variability in non-demented older adults but not the hippocampal volume. A large-scale neuroimaging study showed that thickness measurements are more appropriate to use when assessing neurodegeneration in regions characteristic of AD than volumes because thickness is sufficiently uncorrelated with TIV [18]. In addition, cortical thickness was better than cortical volume or surface area when detecting MCR-s [40]. Consistent with these results, cortical regions associated with gait variability disappeared when GM thickness was changed to volume in the present study. Studies on the association between regional cortical volume and temporal gait variability in older adults without neurological diseases are limited and their results were inconsistent. Beauchet et al. reported that higher temporal gait variability was associated with larger hippocampi [16], whereas other studies could not determine whether temporal gait variability was associated with hippocampal volume [13, 17]. Sakurai et al. reported that the smaller entorhinal cortex was associated with slower dual task gait speed in older adults with MCI but not the hippocampus [17]. This is in line with the results of the current study. A growing body of literature indicates that entorhinal cortex atrophy precedes hippocampal atrophy in pathological aging [41].

This study has several limitations. First, the cross-sectional nature of the current study does not allow for causal interpretation between cortical thinning and higher gait variability. Future longitudinal studies are warranted to examine changes in cortical thickness over time and their relation to gait variability. Second, a once-off assessment of gait variability may not accurately represent one’s true gait variability. The shared neural substrates between gait variability and cognitive function must to be replicated using the gait features obtained over a longer period using a wearable inertia sensor.

## Conclusions

In conclusion, higher gait variability was associated with poorer global cognition in non-demented older adults and cortical thinning of a cluster that includes the inferior temporal, entorhinal, parahippocampal, fusiform, and lingual regions in the left hemisphere. This cluster mediated the association between gait variability and cognitive function.

### Supplementary Information

**Additional file 1: Figure S1.** Cortical thickness and gait variability in older adults with MCI (*n* = 39).

## Data Availability

The datasets used and/or analyzed during the current study are available from the corresponding author on reasonable request.
