# Supplementary figures and images for: Exploring shared neural substrates underlying cognition and gait variability in adults without dementia
Source: Alzheimers Res Ther. 2023 Nov 27;15:206. doi: 10.1186/s13195-023-01354-y (PMC10680297; doi:10.1186/s13195-023-01354-y)

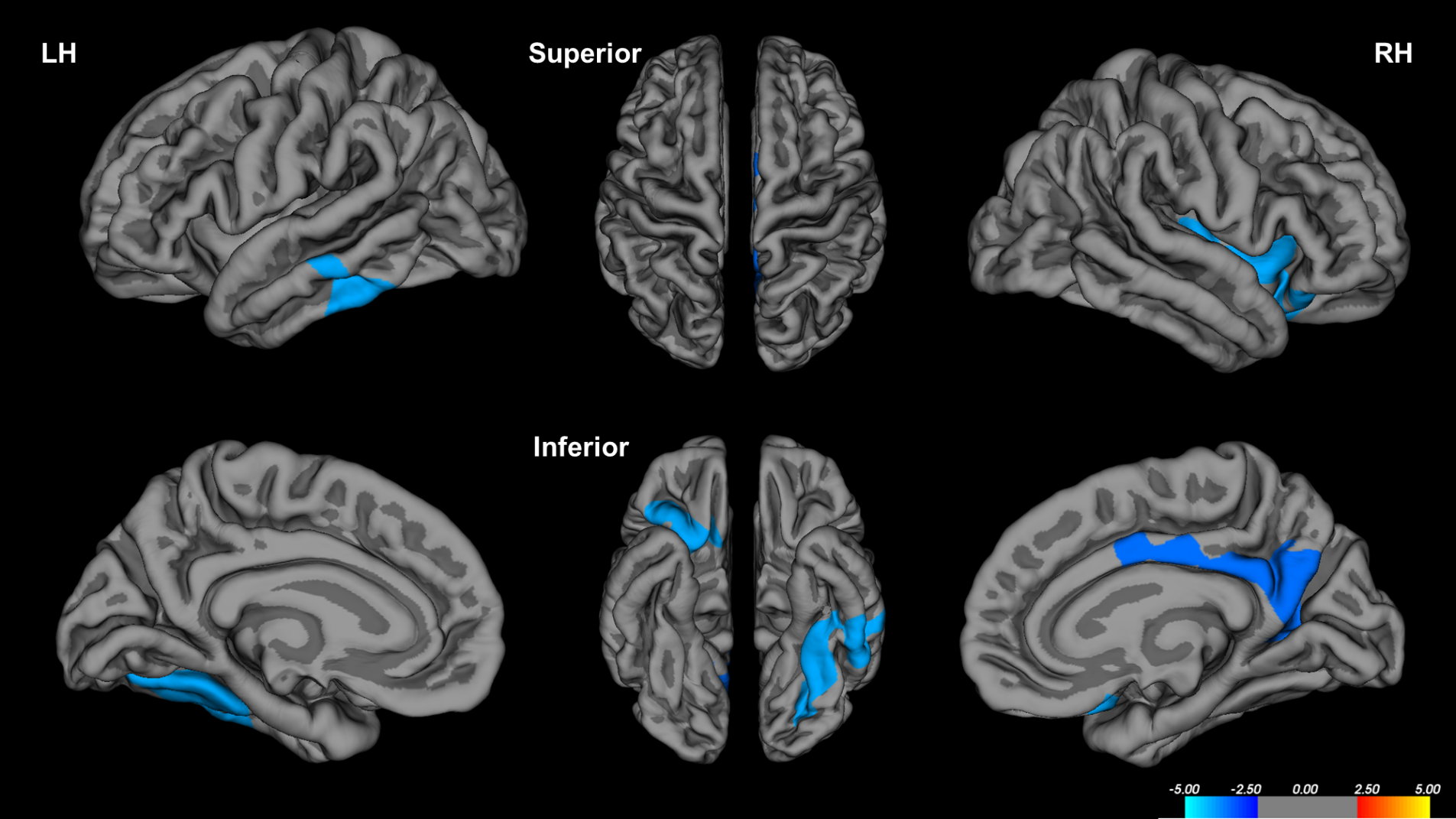


Figure S1. Cortical thickness and gait variability in older adults with MCI (n=39)

Supplement: Supplementary file 1 — Additional file 1: Figure S1. Cortical thickness and gait variability in older adults with MCI (n = 39). [file 13195_2023_1354_MOESM1_ESM.docx]
